# Disease patterns and entities in adult liver consult cases highlight challenging areas in diagnostic hepatopathology practice

**DOI:** 10.1007/s00428-025-04215-1

**Published:** 2025-08-13

**Authors:** Ioanna Maria Grypari, Iakovos Vlachos, Eleni Stoupi, Despoina Karandrea, Despoina Myoteri, Dina Tiniakos

**Affiliations:** 1 Department of Pathology, Aretaieion Hospital, Medical School, National and Kapodistrian University of Athens, 76 Vas. Sofias Avenue, Αthens, 11528 Greece; 2 Department of Pathology, General Hospital of Athens Ippokrateion, Athens, Greece; 3 Translational & Clinical Research Institute, Faculty of Medical Sciences, Newcastle University, Framlington Place, Newcastle upon Tyne, NE2 4HH UK

**Keywords:** Liver biopsy, Hepatopathology, Liver pathology, Consult, Vascular liver disease, Hepatocellular carcinoma, Medical education, Porto-sinusoidal vascular disorder

## Abstract

Difficult liver pathology cases often require expert review for optimal patient care. We reviewed consult cases received in our reference centre aiming to highlight challenging areas in hepatopathology that may benefit from specialist consultation and focused educational activities. We included all primary liver consult cases received between 10/2016 and 12/2022. Data on the sender and aetiology for consultation were collected. Initial and consult reports were screened for adequacy of clinical information, stains performed, final diagnosis and disease grading/staging. We retrieved 219 liver consults, 187 submitted by hepatologists. For medical cases, most common initial diagnoses were non-specific changes, chronic hepatitis and cirrhosis without underlying aetiology. Most common consult diagnoses were vascular disease, primary biliary cholangitis and metabolic dysfunction-associated steatohepatitis (*p* < 0.001). Major change in initial diagnosis was noted in 72 (49%), minor change in 38 (25.9%) and no change in 23 (15.6%) cases. For focal liver lesions (*n* = 72), most common initial diagnoses were hepatocellular carcinoma (HCC), non-specific or metastatic. Most common consult diagnoses were HCC, cholangiocarcinoma or non-neoplastic liver (*p* = 0.033). Major change in initial diagnosis was noted in 30 (41.7%), minor change in 24 (33.3%) and no change in 15 (20.8%) cases. Study of liver consult patterns provides useful information on areas of hepatopathology posing diagnostic difficulty. Most challenging fields are vascular liver disease, interpretation of hepatitic pattern, primary cholangiopathy and classification and subtyping of hepatocellular tumours. These areas can be the subject for future medical educational activities in hepatopathology.

**Supplementary Information:**

The online version contains supplementary material available at 10.1007/s00428-025-04215-1.

## Introduction

Hepatology is a complex field of internal medicine demanding a variety of diagnostic tests, including liver biopsy in challenging cases. Liver biopsy, despite being an invasive procedure, cannot be substituted in these cases by non-invasive techniques [1]. Liver biopsy is indicated for confirmation or ruling out a suspected clinical diagnosis, predictive and prognostic information, monitoring therapeutic response and classifying or subtyping an indeterminate focal liver lesion [1–3].

Interpretation of liver biopsy is challenging, and though it provides significant help in daily hepatology practice, it often poses diagnostic difficulties even among experienced general pathologists. Thus, having reference centres for hepatopathology is key as diagnostic dilemmas can be solved most of the time by a specialized hepatopathologist.

Scarce data exist on which areas of liver pathology are more challenging for diagnosis based on studies of external or extramural consult cases received in tertiary academic centres with hepatopathology service [4–7]. These cases are problematic for general pathologists, causing delays or sometimes misinterpretation of diagnosis, impairing patient management. With the increasing workload in pathology laboratories worldwide, specialist pathologist review is valuable by decreasing time to diagnosis and offering more precise diagnosis, improving the quality of patient management. The few existing studies on second opinion liver pathology are hampered by inter-observer variability as they are either multicentre [8] or they originate from a single centre where the consult cases were evaluated by at least two different hepatopathologists [5–7]. These studies have been conducted in the USA [7, 8] or the UK [5, 6], and there are no data from continental Europe or Australasia. We have evaluated all cases received for second opinion in our reference centre in Greece, reviewed by a single experienced hepatopathologist, aiming to highlight challenging areas in hepatopathology that may benefit from consultation and focused educational activities.

## Materials and methods

We evaluated the reports of all primary liver consult cases received at the Department of Pathology, Aretaieion Hospital between October 2016 and December 2022. All cases were diagnosed by a single specialized academic hepatopathologist (D.T.). Data collected included type of specimen (needle biopsy or resection), type of biopsy (medical or lesional biopsy), case submitter (hepatologist, pathologist or patient), reason for consult or question to consultant pathologist, adequacy of information accompanying each case (clinical, laboratory, imaging), initial diagnosis, adequacy and necessity of initial stains and additional stains performed during consult (histochemical, immunohistochemical), consult diagnosis including underlying disease aetiology and disease grading and/or staging where applicable. The absence of a specific clinical question was interpreted as a request to confirm initial diagnosis.

Agreement with or change of the initial diagnosis by the consulting hepatopathologist was recorded in all cases. Major change was defined as an entirely different diagnosis resulting in alteration of patient management. Minor change was defined as a modification of the initial diagnosis adding prognostic information. In medical liver cases, minor change included adding a histological score (NAFLD activity score/NAS or stage in metabolic dysfunction-associated liver disease-(MASLD) cases [9, 10] or grading inflammatory activity and staging in primary cholangiopathies [11]. In lesional cases, minor changes included hepatocellular carcinoma (HCC) subtyping or morphomolecular classification for hepatocellular neoplasms.

Descriptive statistics were presented as counts and percentages for qualitative data. Quantitative data were described by the mean and standard deviation or median with interquartile range. Groups were compared with Pearson’s chi-square test for qualitative data and the Student’s *t*-test for quantitative data. All tests were two-sided and a *p*-value of 0.05 or less was considered statistically significant. Statistical analyses were performed with the use of SPSS 29.0 software (SPSS Inc., Chicago, USA).

## Results

### Patient demographics and type of specimen

We examined 219 consult reports of liver specimens from 219 patients. There were 112 female patients (51.14%) with median age 53.5 years (range 13–86 years, interquartile range 21) and 107 male patients (48.86%) with median age 56 years (range 17–84 years, interquartile range 23). Information about age was not available in five patients (four female and one male). Patient characteristics according to specimen type are presented in detail in Table 1.

**Table 1:** Patient demographics of submitted consult cases according to type of specimen

	Consults (***n***)	Patient age (years, median ± standard deviation)
Patient numbers		
Total	219	55 ± 15.365
Male	107	56 ± 16.017
Female	112	53.5 ± 14.761
Medical consults	147	51 ± 15.302*
Male	63	48 ± 16.335
Female	80	53 ± 14.216
Focal lesion consults	72	61 ± 13.235*
Male	44	62 ± 11.778
Female	28	59 ± 15.529

^*^p < 0.001

Cases included 176 (80.4%) needle biopsies (134 medical and 42 lesional) and 43 (19.6%) resection specimens (13 medical and 30 lesional). Overall, there were 147 (67.1%) medical (mean age 50.2, range 13–86, interquartile range 20) and 72 (32.9%) lesional (mean age 60.04, range 27–85, interquartile range 19) cases.

Patients with focal liver lesions were 10 years older compared to patients who underwent liver biopsy for medical reasons (*p* < 0.001). In the group of lesional biopsies, male patients were older than female patients (*p* = 0.017), whereas the opposite was observed in medical liver biopsies (*p* = 0.037). No other significant correlation was noticed.

Only three paediatric cases, regarding medical needle biopsies of one 13-year-old female and two 17-year-old males, were studied. Their number was too small to draw meaningful comparisons.

### Submitter of the consults and aetiology of submission

The vast majority of cases, 187 (85.4%), were submitted by hepatologists, 20 (9.1%) were sent by pathologists, 9 (4.1%) by surgeons, and only 2 (0.9%) were re-examined on patient request. The data of the submitters are presented in Supplementary Table 1. Tables 2 and 3 summarize data on the clinical question for each case.

**Table 2:** Specific clinical question to the consultant for medical cases

Specific question	Count (%)
No question or confirmation of diagnosis	74 (50.3)
Autoimmune hepatitis	17 (11.6)
Metabolic dysfunction-associated steatohepatitis	11 (7.5)
Primary biliary cholangitis	11 (7.5)
Aetiology of cirrhosis	9 (6.1)
Drug induced liver Injury	8 (5.4)
Assessment of non-neoplastic liver	6 (4.1)
Autoimmune cholangitis	4 (2.7)
Primary sclerosing cholangitis	3 (2.0)
Wilson disease	2 (1.4)
Vascular disease	1 (0.7)
Rule out malignancy	1 (0.7)

**Table 3:** Specific clinical question to the consultant for focal liver lesions

Specific question	Count (%)
Type of neoplasm *HCC confirmation, HCA vs HCC, FNH vs HCA, dysplastic nodule vs HCC, CC*	35 (48.6)
No clinical question or confirmation of diagnosis	26 (36.11)
Assessment of non-neoplastic liver	5 (6.9)
Rule out malignancy	3 (4.2)
Primary sclerosing cholangitis	1 (1.4)
Type of neoplasm and assessment of non-neoplastic liver	1 (1.4)
Primary biliary cholangitis/Granulomas/Vascular disease	1 (1.4)

*HCC* hepatocellular carcinoma; *HCA* hepatocellular adenoma; *FNH* focal nodular hyperplasia; *CC* cholangiocarcinoma

### Submission data

In medical biopsies, where clinical information is crucial for the correct diagnosis and/or clinical-pathology correlation, 44 (29.9%) were submitted with inadequate information, 56 (38.1%) without any clinical data, and only 35 cases (23.8%) were sent with adequate medical history. In 84 (57.14%) cases, additional data were sent only to the consultant, and in 38 cases (25.85%), extra information was provided following the consultant’s request. For focal hepatic lesions, imaging data were not known in 49 (68.1%) cases. All submission data regarding clinical information, laboratory test results, and imaging findings are summarized in Supplementary Table 2.

### Submitted diagnosis

In 17/219 (7.8%) cases, there was no initial histology report available. From the remaining 202 cases, 55 cases (25.1%) had no specific initial histological diagnosis, and when the results were analyzed separately for medical and lesional biopsies, an inconclusive diagnosis was more frequent in medical cases (38 (25.9%) versus 17 (23.6%), *p* < 0.001). Numbers of all submitted diagnoses are shown in Supplementary Table 3.

### Submitted versus consult diagnosis

Agreement with initial diagnosis and minor or major changes in final diagnosis by the expert consultant are summarized in Table 4. In almost half of all cases (102/219, 46.6%), a major change was proposed after consultation.

**Table 4:** Outcomes of 219 consult diagnoses compared to available initial diagnosis

Consult outcome	Total *n* (%)	Medical *n* (%)	Focal lesion *n* (%)
Agree	38 (17.3)	23 (15.6)	15 (20.8)
Minor change	62 (28.3)	38 (25.9)	24 (33.3)
Major change	102 (46.6)	72 (49)	30 (41.7)
Missing initial report	17 (7.8)	14 (9.5)	3 (4.2)
Total	219 (100)	147 (100)	72 (100)

Table 5 summarizes changes in the diagnosis of medical liver cases compared to the submitted diagnosis. The most common change after consultation was for no specific diagnosis in the initial report. These cases were more frequently re-diagnosed as vascular disease, primary biliary cholangitis (PBC) or steatotic liver disease (SLD), while the chronic cholestatic pattern of injury not otherwise specified (NOS) more frequently changed to PBC or chronic cholestatic pattern with a proposed aetiology. Specific aetiology was assigned in seven cases of cirrhosis submitted as cryptogenic. In a subset of cases submitted as metabolic dysfunction-associated steatohepatitis (MASH), MASH concurrent with autoimmune hepatitis (AIH) requiring immunosuppression was recognized by the consultant, while in five cases initially signed out as SLD, three had MASH.

**Table 5:** Changes in diagnosis of medical liver cases compared to initial diagnosis

**Initial diagnosis**	**Change** in diagnosis (***n***)	**Consult diagnosis** (***n***)
No specific diagnosis	21	Vascular disease (8) PBC (3) Steatotic liver disease (3) Cholestatic pattern (NOS) (2) CHF/ductal plate malformation (1) DILI (1) Ciliopathy (1) HBV infection (1) Fibrosis/cirrhosis with underlying aetiology (1)
Chronic hepatitis	13	MASH (3) PBC (2) AIH (2) Vascular disease (2) Cholestatic disease (2) PSC (1) DILI (1)
Cirrhosis without underlying aetiology	7	MASH (2) Non-specific changes (1) Concurrent MASH and AIH (1) AIH (1) Granulomatous hepatitis (1) HCV hepatitis (1)
MASH	5	Concurrent MASH and AIH (3) Fibrosis/cirrhosis, with underlying aetiology (1) AIH (1)
Steatotic liver disease	5	MASH (3) Vascular disease (1) AIH-PSC variant (1)
Cholestatic pattern, NOS	4	PBC (2) Proposed aetiology (2)
Drug induced liver injury	4	AIH (1) Granulomatous hepatitis (1) Chronic hepatitis with proposed aetiology (1) MASLD (1)
Primary sclerosing cholangitis	4	DILI (1) Vascular disease (1) Amyloidosis (1) PSC-AIH variant (1)
Acute hepatitis	1	AIH
Vascular disease	1	No specific changes
NRH	1	Sclerosing cholangitis
Biliary micro-hamartoma	1	CHF/ductal plate malformation
Sclerosing cholangitis	1	DILI

*AIH* autoimmune hepatitis; *CHF* congenital hepatic fibrosis; *HBV* hepatitis B virus; *HCV* hepatitis C virus; *DILI* drug-induced liver injury; *MASLD* metabolic dysfunction-associated steatotic liver disease; *MASH* metabolic dysfunction-associated steatohepatitis; *NOS* not otherwise specified; *NRH* nodular regenerative hyperplasia; *PBC* primary biliary cholangitis; *PSC* primary sclerosing cholangitis

Among the three paediatric medical biopsies, the initial report was missing in one case; in one case of acute cholestatic hepatitis, possible autoimmune aetiology was proposed after consultation, while in one case of vascular liver disease, the consult report agreed with the initial diagnosis.

Table 6 summarizes changes in the diagnosis of focal liver lesions compared to submitted diagnosis. Cases with no specific diagnosis (*n* = 12) were the most frequently altered by the consultant, changing more often to focal nodular hyperplasia (FNH), HCC or non-tumour diagnosis. Among four cases initially submitted as HCC, one was re-classified as a cholangiocarcinoma, changing dramatically patient treatment, while the remaining were indeed HCC but of specific subtypes with valuable management implications.

**Table 6:** Changes in diagnosis of focal liver lesions compared to initial diagnosis

**Initial diagnosis**	**Change in diagnosis** **(*n*)**	**Consult diagnosis** (***n***)
No specific diagnosis	12	HCC (5) Vascular disease/FNH (3) No tumour (3) (2 PBC & 1 MASH) HUMP/atypical HCN (1)
HCC NOS	4	HCC with microvascular invasion (2) CCA (1) Fibrolamellar HCC (1)
HCA	3	HCC (2) HUMP/atypical HCN (1)
Metastatic carcinoma	2	HCC (2)
CCA	1	HCC
Haematopoietic tumour	1	Inflammatory pseudotumour
Benign vascular tumour	1	Angiosarcoma
Combined HCC/CC	1	HCC
Missing initial report	1	FNH

*HCC* hepatocellular carcinoma; *FNH* focal nodular hyperplasia; *CCA* cholangiocarcinoma; *AIH *autoimmune hepatitis;* MASH* metabolic dysfunction-associated steatohepatitis; *HCA* hepatocellular adenoma; *HUMP/HCN* hepatocellular neoplasm of uncertain malignant potential/atypical hepatocellular neoplasm; *NOS* not otherwise specified; *NRH* nodular regenerative hyperplasia

### Adequacy of ancillary stains

In 16.9% of all cases, no additional stain was needed for consult diagnosis. However, in 100 (68%) medical cases, ancillary stains used to facilitate initial diagnosis were inadequate; therefore, additional stains were performed by the consultant to establish diagnosis. In contrast, in 19 (12.92%) medical cases, unnecessary stains were initially performed. Forty-nine (68.1%) lesional cases were submitted with inadequate stains, while in 31 (43.1%) cases, unnecessary stains were initially done. Unnecessary stains were more commonly performed for focal liver lesions compared to medical cases (*p* < 0.001), while medical cases had more frequently suitable special stains performed at initial diagnosis (*p* = 0.007).

Additional stains during consult were more frequently both immunohistochemical and histochemical (49.8%), while only immunohistochemical (22.8%) or only histochemical (6.4%) were performed in a minority of cases.

Supplementary Table 4 shows useful additional histochemical and immunohistochemical stains performed during consult to facilitate diagnosis of medical liver cases. The common panel of histochemical stains for evaluating non-neoplastic liver disease (the “liver panel”) was applied. Keratin 7 (K7) immunostain was performed routinely to highlight ductular reaction, periportal K7-positive intermediate hepatocytes indicative of chronic cholestasis and more centrally located K7-positive hepatocytes indicative of parenchymal ischaemia. CD34 immunostain was applied to assess the presence of sinusoidal capillarization in vascular disease, while glutamine synthetase was used to highlight centrilobular areas in cases where these were obscured.

Supplementary Table 5 shows useful additional histochemical and immunohistochemical stains performed during consult to facilitate diagnosis of lesional cases of hepatocellular origin. The most useful, albeit commonly omitted at initial diagnosis, histochemical stain was reticulin to highlight the thickness of hepatic trabeculae, nodular morphology and loss of reticulin framework indicative of malignancy. In addition to reticulin, immunohistochemical stains aiding the differential diagnosis between high-grade dysplastic nodule and well-differentiated HCC included glypican 3, glutamine synthetase and heat-shock protein 70. “Geographical pattern” of glutamine synthetase expression was pivotal for the diagnosis of FNH.

## Discussion

We have shown that the study of liver consult patterns may provide useful information on areas of hepatopathology posing diagnostic difficulty. In medical cases, the most common diagnostic challenges are vascular liver disease, interpretation of hepatitic pattern of injury and recognition of a primary cholangiopathy, while in tumour cases, these include mainly classification and subtyping of hepatocellular tumours.

In the past, before the advent of successful antiviral treatment and non-invasive techniques for assessing liver fibrosis that decreased the need for liver biopsy [1, 12], discrepancies between initial and consult liver cases were related to the assessment of necroinflammatory activity and fibrosis in viral hepatitis [13–15]. In a study on liver biopsies with chronic hepatitis C, interobserver agreement between hepatopathologists and community pathologists was satisfactory, but the most ambiguous field was determination of fibrosis stage, mainly in cases with short liver cores [16]. These older studies, although focused on a specific topic of liver pathology, reflect the timeless need for specialized evaluation in liver pathology.

Discrepancy between initial and consult diagnosis is common in studies similar to ours. This is mainly attributed to the complex nature of hepatopathology with overlapping patterns of hepatic injury. Moreover, the number of diagnostic liver biopsies has decreased over the years as non-invasive methods for assessing fibrosis gained ground [12, 17]. Thus, the number of liver cases general pathologists sign out routinely is highly variable. It is possible that pathologists are not exposed to the whole spectrum of liver pathology unless they are trained in a tertiary academic hepatology centre, which is not commonly the case, especially in small countries like Greece.

Hepatologists are the most common medical specialists submitting medical and lesional liver cases for second opinion, while only a few cases get a second opinion at a pathologist’s initiative. This is in contrast to previous studies, where most of the cases were submitted by a pathologist [6, 8]. This could be attributed to different health care system structures, as in Greece liver biopsies usually are not performed in the centre where the patient will be treated.

Hepatopathology is an excellent paradigm of evidence-based medicine [18], as the proper diagnosis and subsequent patient management require clinico-pathological correlation. However, clinical, laboratory and imaging information are not always provided when a liver sample is received, as it is demonstrated in our cohort, complicating the pathologist’s work. A specialized hepatopathologist is aware of the utility of clinical history when assessing a liver tissue sample and will ask for additional information when needed in order to correlate the pattern of liver injury with the clinical context [19]. It is indeed recommended that relevant clinical data should not be missing from the specimen submission form, as their presence saves time for all members of the medical team and especially for pathologists.

A major change in the histological diagnosis altering patient management occurred in more than half of submitted cases (51%), more frequently in medical cases (55%) compared to lesional (30%). In medical cases, the most under-recognized entity was vascular disease, a spectrum of blood flow abnormalities which in our study included nodular regenerative hyperplasia (NRH), porto-sinusoidal vascular disorder (PSVD) and toxic microvascular injury (sinusoidal obstruction syndrome). The histological changes in these cases were initially interpreted mainly as non-specific or as chronic hepatitis. This is not surprising as histological changes in the spectrum of PSVD are subtle and the entity is newly recognized [20], while NRH diagnosis requires reticulin stain that is not often performed by general pathologists. Another difficult to diagnose biliary entity was PBC and was often described as non-specific changes, cholestasis of unknown aetiology or chronic hepatitis. This difficulty at first diagnosis could be attributed to the focality of biliary that is common in PBC and the lack of typical histological features, such as destructive granulomatous cholangitis with bile duct loss [21]. These changes were evident in deeper tissue sections assessed by the consult pathologist. Other diagnostic features, which require interpretation in a specific biochemical/serological context, are parenchymal necroinflammatory foci, interface inflammatory activity lacking plasmacytes and NRH [21]. MASH was also an under-recognised diagnosis initially confused with chronic hepatitis of other aetiology or not recognized in the cirrhotic stage with no comment on underlying aetiology. By definition, MASH diagnosis requires the presence of steatosis, hepatocellular injury in the form of hepatocyte ballooning and lobular inflammation [22]. The identification of ballooned hepatocytes is a matter of disagreement even among expert hepatopathologists [23], although inter-observer variability significantly decreases following consensus definitions and harmonization. Ballooning is one of the hallmarks of steatohepatitis, and its presence is crucial for MASH diagnosis, required for enlisting patients in clinical therapeutic trials and highlighting SLD patients who need specialized multidisciplinary care and have worse prognosis for cardiovascular or liver complications, such as cirrhosis and HCC [24].

Most focal liver lesion specimens submitted for consultation were inadequately interpreted by general pathologists and were signed out as inconclusive. Cases that fell into this category were mainly HCC and FNH. The former was very often misclassified as cholangiocarcinoma or as hepatocellular adenoma (HCA) and less frequently as metastatic carcinoma. Distinction between HCC and cholangiocarcinoma determines patients’ management with completely different therapeutic options for each entity, as cholangiocarcinoma may harbour targetable mutations or fusions [25]. Specific subtypes of HCC with prognostic implications, such as fibrolamellar HCC [26] or with the presence of beta-catenin gene mutations that may affect therapeutic management [27, 28], were often missed in the initial report. Additionally, a case of angiosarcoma was considered a benign vascular tumour, a misdiagnosis with significant prognostic and therapeutic implications. The complexity of histopathology patterns of angiosarcoma, combined with its rarity, challenges even expert hepatopathologists, especially in needle biopsy material [27]. Thus, raising awareness and focused training for the recognition of these rare and aggressive tumours is pivotal for their correct identification.

In many cases, insufficient or redundant ancillary stains were initially performed, highlighting an emerging difficulty for pathology laboratories, balancing constantly increasing workload with high-quality standards for tissue sample assessment by the pathologist. Targeted and efficient use of resources, including special stains, reduces unnecessary workflow and retains a reasonable turnaround time for a precise histopathology report [28].

In conclusion, our study indicates that there are specific areas of hepatopathology which demand an expert’s opinion. These include vascular liver disease, primary cholangiopathies and MASH, while in neoplastic liver pathology, HCC and its subtypes, FNH, cholangiocarcinoma and vascular tumours are often problematic to identify. Identification of these areas may guide future continuing education in liver pathology.

## Supplementary Information

Below is the link to the electronic supplementary material.

ESM1 (DOCX 24.6 KB)

## Data Availability

All research data and material will be made available upon request. Most data are included in the main manuscript and supporting files.
