# Supplementary material for: Disease patterns and entities in adult liver consult cases highlight challenging areas in diagnostic hepatopathology practice
Source: Virchows Arch. 2025 Aug 13;487(6):1291–8. doi: 10.1007/s00428-025-04215-1 (PMC12748106; doi:10.1007/s00428-025-04215-1)
Supplement: Supplementary file 1 — (DOCX 24.6 KB) [file 428_2025_4215_MOESM1_ESM.docx]

**Supplementary Table 1.** Source of submitted consults (n=219) according to consult type

| SOURCE | COUNT |
| --- | --- |
| Hepatologist | **187** |
| Medical | 135 |
| Focal lesion | 52 |
|  |  |
| Pathologist | **20** |
| Medical | 10 |
| Focal lesion | 10 |
|  |  |
| Surgeon | **9** |
| Medical | 0 |
| Focal lesion | 9 |
|  |  |
| Patient | **2** |
| Medical | 1 |
| Focal lesion | 1 |
|  |  |
| Unknown submitter | **1** |
| Medical | 1 |
| Focal lesion | 0 |

**Supplementary Table 2.** Relevant information received with the initial report according to type of case.

|  | Adequate  n (%) | Inadequate  n (%) | None  n (%) | Missing initial report  n (%) |
| --- | --- | --- | --- | --- |
| ALL CASES (n=219) |  |  |  |  |
| Clinical information | 49 (22.4) | 63 (28.8) | 90 (41.1) | 17 (7.8) |
| Laboratory tests | 27 (12.3) | 49 (22.4) | 126 (57.5) | 17 (7.8) |
| Imaging findings | 27 (12.3) | 15 (6.8) | 160 (73.1) | 17 (7.8) |
|  |  |  |  |  |
| MEDICAL (n=147) | | | | |
| Clinical information | 35 (23.8) | 44 (29.9) | 56 (38.1) | 12 (8.2) |
| Laboratory tests | 26 (17.7) | 42 (28.6) | 67 (45.6) | 12 (8.2) |
| Imaging findings | 16 (10.9) | 8 (5.4) | 111 (75.5) | 12 (8.2) |
|  |  |  | |  |
| LESIONAL (n=72) | | | |  |
| Clinical information | 14 (19.4) | 19 (26.4) | 34 (47.2) | 5 (6.9) |
| Laboratory tests | 1 (1.4) | 7 (9.7) | 59 (81.9) | 5 (6.9) |
| Imaging findings | 11 (15.3) | 7 (9.7) | 49 (68.1) | 5 (6.9) |

**Supplementary Table 3.** Consult cases according to type and presence of submitted initial histological diagnosis.

| CONSULT CASES | ALL  n (%) | MEDICAL  n (%) | FOCAL LESION  n (%) |
| --- | --- | --- | --- |
| Total number | 219 | 147 | 72 |
| Submitted/suggested diagnosis | 147 (67.1) | 96 (65.3) | 51 (70.8) |
| No conclusive diagnosis in the report | 55 (25.1) | 38 (25.9) | 17 (23.6) |
| Missing initial histology report | 17 ( 7.8) | 13 ( 8.8) | 4 ( 5.6) |

**Supplementary Table 4.** Ancillary histochemical and immunohistochemical stains aiding the diagnosis of medical liver cases

| Histochemical stains (the “liver panel”) |
| --- |
| Reticulin |
| PAS/ PAS-diastase |
| Collagen stain (Masson trichrome, Sirius/picrosirius red, van Gieson, etc) |
| Orcein |
| Perls |
| Immunohistochemical stains |
| Keratin 7  CD34  Glutamine synthetase |

**Supplementary Table 5.** Ancillary histochemical and immunohistochemical stains aiding the diagnosis of hepatocellular tumours in lesional liver cases

| Histochemical stains |
| --- |
| Reticulin |
| Immunohistochemical stains |
| Glypican 3 |
| Glutamine synthetase |
| Heat-shock protein 70 |
